# The use of human thermal modelling to assess performance during extreme exposures

**DOI:** 10.1186/2046-7648-4-S1-A13

**Published:** 2015-09-14

**Authors:** Marshall Nuckols, Eugene Wissler, Pratibha Sinha, Gary N Proulx

**Affiliations:** 1 Duke University, Mechanical Engineering Department, Durham, NC, USA; 2 University of Texas, Department of Chemical Engineering, Austin, Texas, USA; 3 Navy Clothing and Textile Research Facility, Natick, MA, USA; 4 U.S. Army Natick Soldier R&D Center, Natick, MA, USA

## Introduction

Clothing is an important factor in human response to thermal stress, but there is often a large gap between manikin studies and prediction of human behaviour under actual conditions. Although that gap can be bridged with experimental studies involving human subjects, such studies are difficult to execute and costly to perform. Moreover, human studies cannot be performed under life-threatening conditions. An alternative approach is to predict human behaviour using a thermal model. While that approach greatly reduces the need to conduct experiments with human subjects, validation of a model for a particular application still requires human studies. In this paper we discuss several applications of a human thermal model. One involves an investigation of systems for electrically heating divers during long-term immersion in cold water. Another describes a system currently under development that will allow Navy officers to assess the adequacy of available garments for a particular mission.

## Methods

The human thermal model employed in these studies has evolved over half a century. Human geometry in the current model is represented by 21 cylindrical elements. Temperatures, physiological properties (metabolic and perfusion rates), and physical properties (density, specific heat and thermal conductivity) are defined in 169 small regions within each major element, and up to 6 additional shells (72 additional small regions) represent clothing. Pennes bioheat equation is solved using a two-dimensional finite-difference technique. Physiological control functions are based on the results of physiological studies, and are not simply parametric variables defined to obtain good agreement between computed and measured thermal variables [1].

## Results

Thermal properties of the divers' drysuits were measured using a submersible manikin at the Navy Clothing and Textile Research Facility (NCTRF) in Natick, MA. Cold water trials were then conducted at the Navy Experimental Diving Unit in Panama City, FL with seven test subjects wearing the characterized drysuits to validate the model for this application. The validated model was then used to evaluate different electrical heating arrangements, and predicted behaviour was compared with actual performance of several suits during a total of 20 manned dives.

## Discussion

The combination of manikin testing in combination with simulations using a human thermal model has been shown to be an effective approach to most effectively integrate active heating in cold-water diving garments. A similar approach can also be used to predict the thermal status of personnel while operating in any number of stressful applications, or to create a design tool for developing garments to meet those thermally stressful applications. By utilizing a human thermal model, mission simulations can predict thermal performance in extreme environments without the risk and high cost of exposing human subjects to potentially life-threatening conditions.

## Conclusion

Mathematical human thermal models can be used in various practical ways that have been largely underutilized. Applications require close cooperation between modellers, physiologists, and end users. Our study demonstrates that those efforts yield valuable results.

## References

[B1] Wissler EH Whole-body human thermal modelling - an alternative to immersion in cold water and other unpleasant endeavours J Heat Trans 2012 134

